# Validation of an overnight wireless high-resolution oximeter for the diagnosis of obstructive sleep apnea at home

**DOI:** 10.1038/s41598-022-17698-8

**Published:** 2022-09-07

**Authors:** Rosa Hasan, Pedro Rodrigues Genta, George do Lago Pinheiro, Michelle Louvaes Garcia, Paula Gobi Scudeller, Carlos Roberto Ribeiro de Carvalho, Geraldo Lorenzi-Filho

**Affiliations:** 1 Laboratorio do Sono, LIM 63, Instituto de Psiquiatria (IPq), Hospital das Clínicas HCFMUSP, Universidade de Sao Paulo, Sao Paulo, SP Brazil; 2 Laboratorio do Sono, LIM 63, Divisão de Pneumologia, Instituto do Coraçao, InCor, Hospital das Clínicas HCFMUSP, Universidade de Sao Paulo, Eneas de Carvalho Aguiar 44, 8º andar, Sao Paulo, SP 05403-900 Brazil; 3 Divisão de Pneumologia, Instituto do Coraçao, InCor, Hospital das Clínicas HCFMUSP, Universidade de Sao Paulo, Sao Paulo, SP Brazil

**Keywords:** Diseases, Medical research

## Abstract

Obstructive sleep apnea (OSA) is extremely common and has several consequences. However, most cases remain undiagnosed. One limitation is the lack of simple and validated methods for OSA diagnosis at home. The aim of this study was to validate a wireless high-resolution oximeter with a built-in accelerometer linked to a smartphone with automated cloud analysis (Biologix) that was compared with a home sleep test (HST, Apnea Link Air) performed on the same night. We recruited 670 patients out of a task force of 1013 patients with suspected OSA who were referred to our center for diagnosis. The final sample consisted of 478 patients (mean age: 56.7 ± 13.1 years, mean body mass index: 31.9 ± 6.3 kg/m^2^). To estimate the night-to-night OSA severity variability, 62 patients underwent HST for two consecutive nights. The HST-apnea–hypopnea index (AHI) and the Biologix-oxygen desaturation index (ODI) was 25.0 ± 25.0 events/h and 24.9 ± 26.5 events/h, respectively. The area under the curve—sensibility/specificity to detect at least mild (HST-AHI > 5), moderate-to-severe (HST-AHI > 15), and severe OSA (HST-AHI > 30) were (0.983)—94.7/92.8, (0.986)—94.8/93.9, and (0.990)—95.8/94.3, respectively. The limits of agreement originating from the Bland–Altman plot and the correlation between HST-AHI and Biologix-ODI were lower than the night-to-night HST-AHI variability (25.5 and 34.5 events/h, respectively, *p* = 0.001). We conclude that Biologix is a simple and reliable technique for OSA diagnosis at home.

## Introduction

Obstructive sleep apnea (OSA) is characterized by repetitive episodes of complete (apnea) or partial (hypopnea) upper airway obstruction, leading to intermittent hypoxia and fragmented sleep^1^. OSA is extremely common in the general population and is associated with a variety of symptoms such as habitual snoring, poor and non-restoring sleep, excessive daytime sleepiness, and fatigue, which negatively impact patients’ quality of life^2–4^. Untreated OSA is independently associated with cardiovascular diseases such as hypertension, arrhythmias, coronary artery disease, stroke, and cardiovascular death^5–8^. Despite this evidence, OSA remains largely underdiagnosed^9,10^.

The awareness that OSA is poorly recognized has prompted the popularization of diagnostic methods that are simpler than full polysomnography (PSG). Although PSG is considered the gold standard method, it is a barrier to massive testing because it must be performed at the sleep laboratory under supervision. It is now well established that the home sleep test (HST), which monitors a limited number of respiratory channels at home, performs as well as PSG in patients with suspected OSA^11,12^ The main parameter that determines the presence and severity of OSA is the apnea–hypopnea index (AHI). The AHI expresses the number of apneas plus hypopneas divided by the number of hours of sleep or registered hours, in the case of PSG or HST, respectively^1^. Apneas and hypopneas are typically associated with transitory falls in oxygen saturation. Because the oxygen signal is more robust than the respiratory flow signal, the associated level of oxygen desaturation is used as a criterion for hypopnea definition (minimal of 3% or 4%) and heavily influences hypopnea scoring^13^. Therefore, the oxygen desaturation index (ODI) is highly correlated with AHI^14^. There is also recent evidence that parameters derived from the oxygen signal, such as the hypoxic burden, are better predictors of cardiovascular mortality than AHI^7,14^. In this context, high-resolution oximetry is a simple and attractive technique for OSA diagnosis at home. Simpler OSA diagnostic methods, which can be easily repeated at home, also have the potential to address a well-reported but largely ignored phenomenon in clinical practice, which is OSA severity night-to-night variability^15^. However, the performance of oximeters is variable and dependent on the technology used, and the method is not disseminated and is usually considered a screening diagnostic tool^16^.

We recently validated a new high-resolution wireless oximeter with a smartphone application and an automated cloud algorithm for the detection of oxygen desaturation, described herein as overnight digital monitoring (ODM-Biologix)^14^. However, this study was performed in a controlled environment. In the present study, we hypothesized that ODM-Biologix is an accurate technique for the diagnosis of mild, moderate to severe, and severe OSA at home. To this end, we compared ODM-Biologix with HST performed simultaneously in patients with suspected OSA. We also performed HST in a subgroup of patients during two consecutive nights to determine the night-to-night HST-AHI variability. We hypothesized that the variability between HST-AHI and ODM-ODI would not differ from HST-AHI variability.

## Methods

### Patients

We invited patients with suspected OSA that participated in a fast-track OSA diagnosis task force. Briefly, the task force consisted of patients who were waiting for sleep studies for long periods (mostly > 1 year) at the public health care system of São Paulo city were referred for HST at the Heart Institute, Hospital das Clinicas, University of Sao Paulo, Brazil. The local ethics committee (Comissão de Ética para Análise de Projetos de Pesquisa do HCFMUSP—CAPPesq) approved the protocol (SDC 5039/20/068), and informed consent was obtained from each participant. The study has been performed in accordance with the Declaration of Helsinki.

### Sleep studies

All patients participating in the fast-track HST task force were evaluated in groups of 10–15 patients per day at the Heart Institute. Each day, the first seven patients who arrived at the sleep clinic for the HST task force were invited to participate in the present study. The patients filled out a standardized questionnaire, underwent a standardized physical exam, and took home the Biologix oximeter and an ApneaLink Air device with the following channels: effort belt, nasal cannula, and oximeter. Because several patients in the public health care system are of low income and do not have either a smartphone or stable internet connection, patients who agreed to participate received a smartphone with an internet connection that already had the Biologix App installed. We instructed the patients to use the Biologix wireless oximeter in a finger of the same hand as the HST oximeter. All patients were trained by institutional videos on how to use the Apnea Link and Biologix devices and received personalized instructions on how to perform the sleep test. Patients were asked to turn on the devices immediately before bedtime and to turn them off the following morning upon waking up.

**Figure 1:**
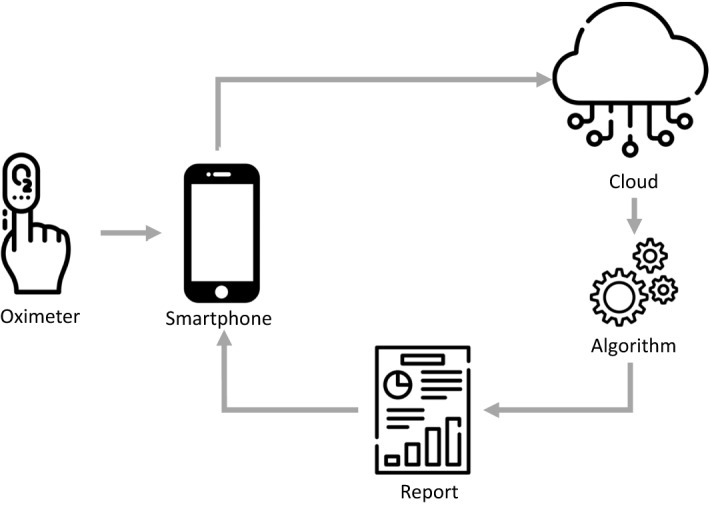
Image descripting the Biologix system. The wireless oximeter connects via Bluetooth to the smartphone Biologix app. An algorithm automatically analyzes the data and the send report to the patient smartphone.

All HST studies were scored manually by one independent certified technician and revised by a sleep medicine physician blinded to the ODM results. Hypopnea was defined as a peak signal excursion drop ≥ 30% of the pre-event baseline nasal pressure signal lasting ≥ 10 s associated with a ≥ 4% reduction in SpO_2_^17^. Mild, moderate, and severe OSA was defined according to the current standards (5 ≤ AHI < 15, 15 ≤ AHI < 30, and AHI ≥ 30 events/h, respectively). The Biologix oximeter (Oxistar™) firmware acquired 100 samples per second, generating a beat-to-beat raw data of SpO_2_ with a resolution of 0.1%. In addition, artifacts were identified as single-beat values that were changed by more than 18%/s during a rising or 6%/s during a falling from the previous beat. Finally, a moving average of four cardiac beats was applied. Data from Oxistar™ are transferred via the smartphone app to the cloud and automatically analyzed using a proprietary algorithm, resulting in the number of desaturations per recording hour (Fig. 1). The ODI results from ODM expressed the number of desaturations per valid recording time and were automatically analyzed using the 4% desaturation criterion. HST recordings with technical problems, including valid register time < 4 hs, loss of signals that compromised a proper conclusion according to the sleep specialist analysis were invited to repeat the study. We excluded from the final analysis recordings with more than 1-h difference between Apnea Link and Biologix. In addition, we invited a convenience sample of patients to perform HST for two consecutive nights to evaluate the night-to-night HST variability.

### Statistical analysis

Continuous data are expressed as the mean ± standard deviation or the median (interquartile range) when appropriate. Bland–Altman plots were used to assess the agreement between HST-AHI versus ODM-ODI and HST-AHI night 1 versus night 2. Student´s unpaired t-test was used to compare the difference in the bias observed in the two Bland–Altman plots (HST-AHI minus ODM-ODI) and (HST-AHI night 1 minus night 2). To compare the variability in the dispersion (95% confidence intervals [CIs]) between the same two Bland–Altman plots, Levene’s test was used. The intra-class correlation coefficient (ICC) and Spearman´s correlation were used to assess the relationship between ODM and HST. The diagnostic performance for each HST cut-off (AHI = 5, 15, and 30 events/h) was assessed by means of sensitivity, specificity, positive predictive value (PPV), negative predictive value (NPV), positive likelihood ratio (LR+), negative likelihood ratio (LR−), accuracy, and area under the receiver operating characteristic curve (AUC).

## Results

**Figure 2:**
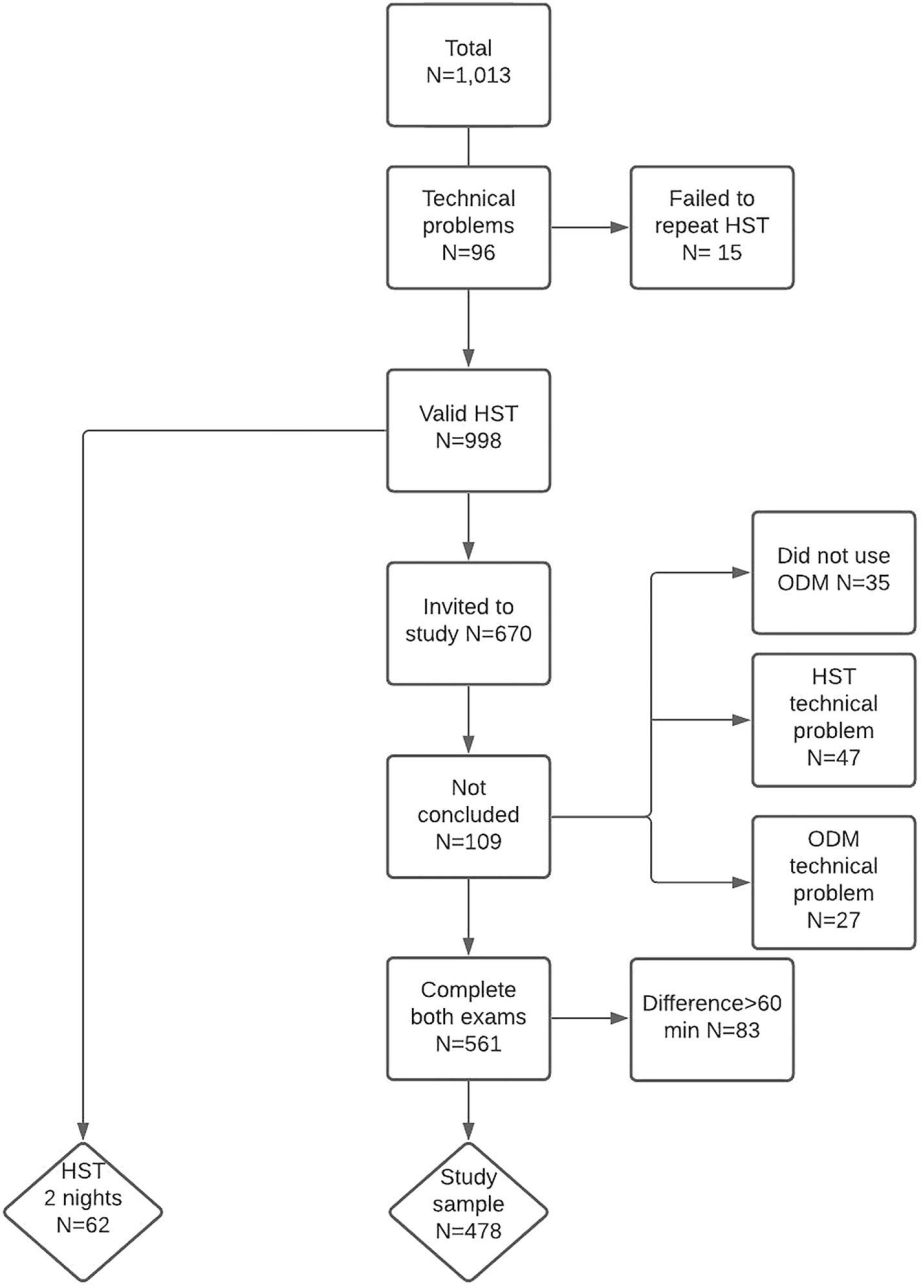
Participant flow diagram. N = number of patients.

A total of 1013 patients underwent HST, 96 patients had to repeat HST due to technical problems, and 15 patients failed to return for the scheduled HST. Therefore, 998 patients underwent a valid HST in the fast-track HST task force. We invited 670 patients to perform Biologix studies; however, 47 had HST technical problems, 27 had technical problems with the ODM study, and 35 failed to use the Biologix device. Therefore, 561 patients completed both the HST and ODM studies. We excluded 83 patients because they had more than one hour of difference between the HST and ODM studies. The final sample comprised 478 patients. To determine the night-to-night HST variability, 62 patients underwent two valid HST tests (Fig. 2).

**Figure 3:**
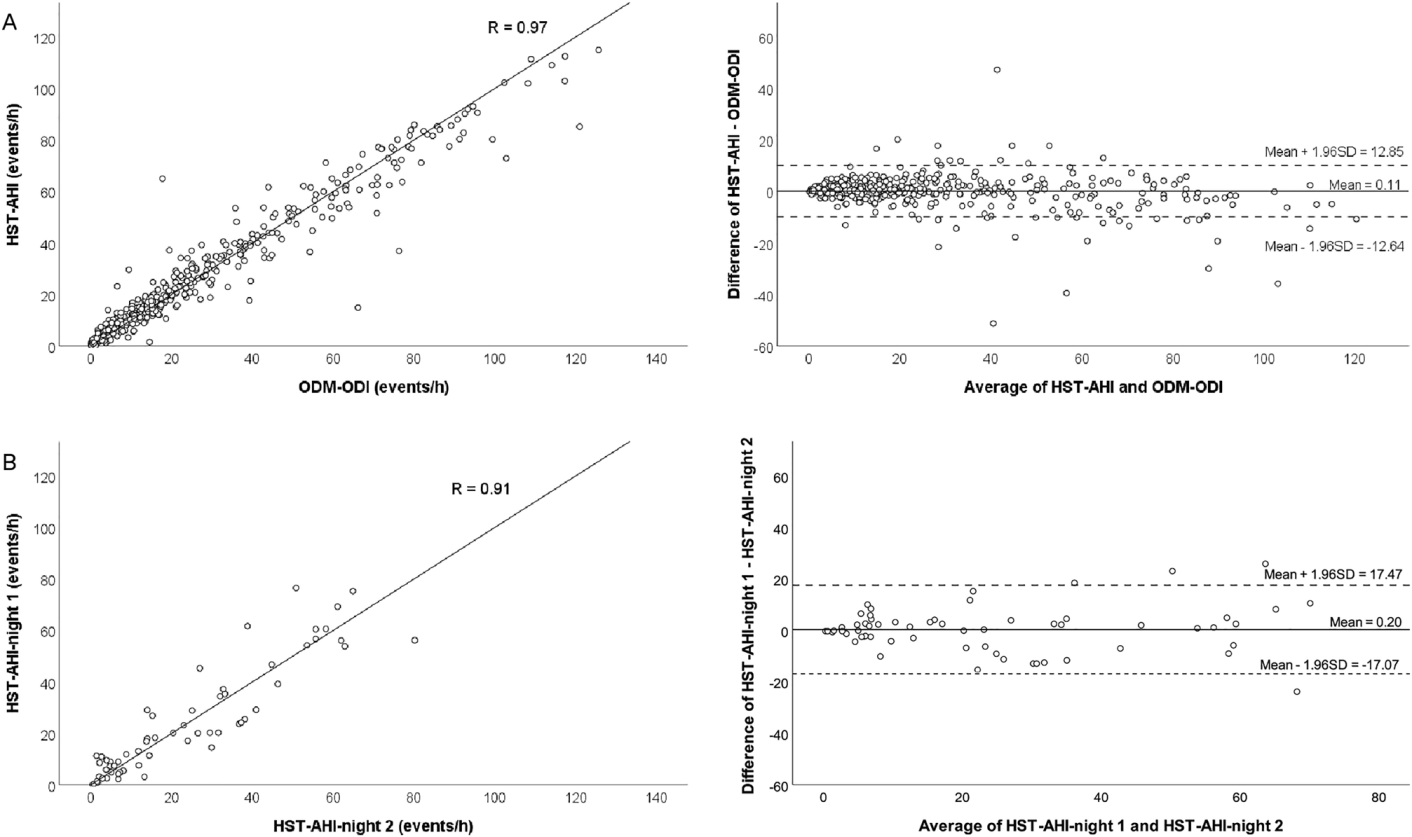
Scatter plots and Spearman’s correlation (r) and Bland–Altman plots comparing (**A**) ODM-ODI and AHI-HST, (**B**) Night 1 and Night 2 HST-AHI.

**Figure 4:**
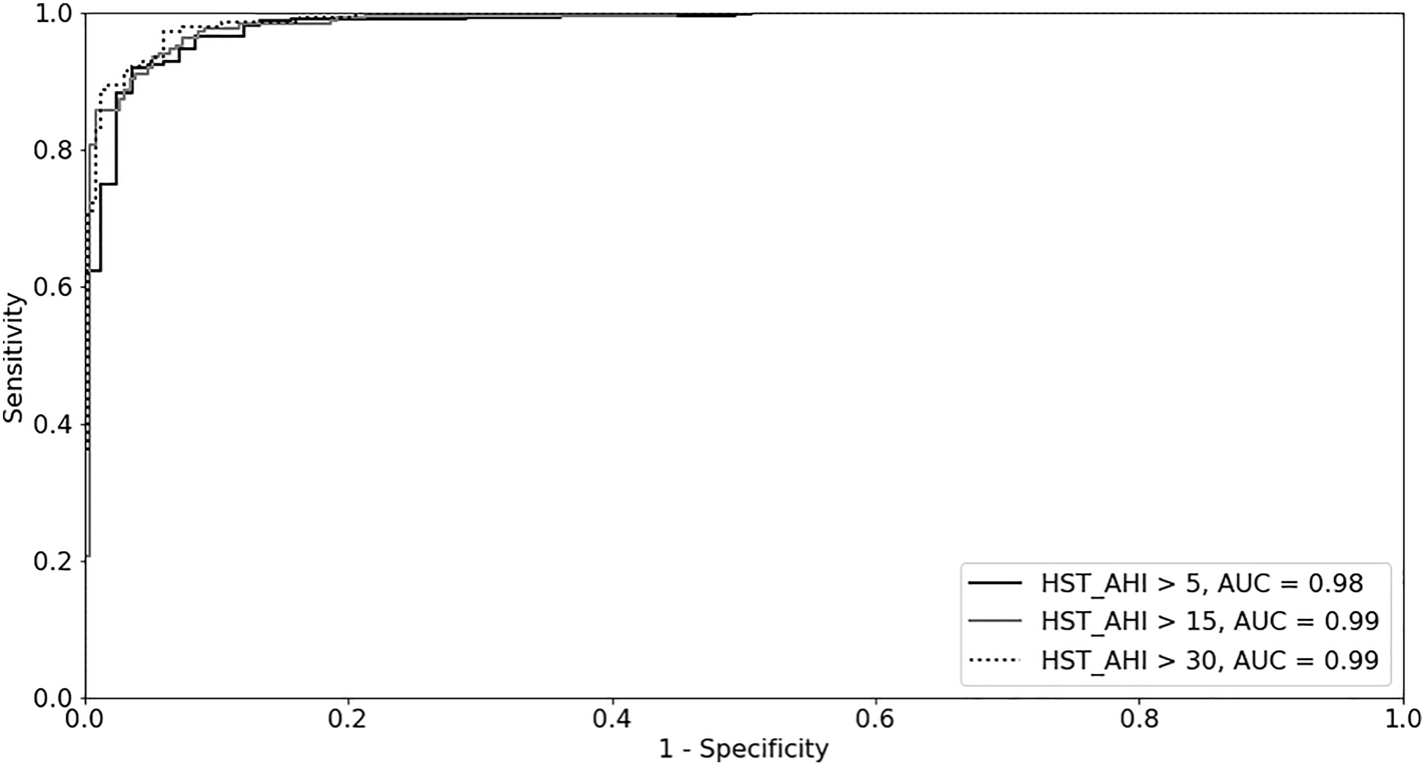
Receiver-operator characteristic curves showing that ODI > 5, > 14, and > 25 events/h were the best cut-offs for detecting at least mild, moderate to severe, and severe OSA, respectively, as evaluated by HST-AHI. The AUC for the ODI cut-offs of > 5, > 14, and > 25 events/h were 0.983, 0.986, and 0.990, respectively.

The population consisted mostly of middle-aged obese adults, frequently referring to excessive daytime somnolence and other comorbidities (Table 1). The characteristics of the total population that participated in the fast-track HST and the patients who participated in the present study were similar (Table 1). Among the entire population and the study sample 17.5% and 17.5% had no OSA (AHI < 5 events/h), 28.0% and 30.9% had mild OSA, 24.4% and 21.9% had moderate OSA, 30.1% and 29.7% had severe OSA. The total register time from HST and ODM were 437.3 ± 72 and 431 ± 74.2 min. The HST and ODM ODI were 24.2 ± 24.8 and 24.9.7 ± 26.5 events/h, the mean SaO_2_ was 92.3 ± 3.1 and 93.2 ± 3.1%, and the minimal SaO_2_ was 80.3 ± 7.0 and 81.0 ± 7.0%, respectively. The HST night-to-night variability is presented in Table 2 and Fig. 3B. The mean bias between HST-AHI and ODM-ODI was very small and similar to the mean bias between night-to-night HST variability (0.11 vs 0.20). The 95%CI originating from the Bland–Altman plot that compared HST-AHI versus ODM-ODI was lower than the HST night-to-night variability (25.5 and 34.5 events/h, respectively, *p* = 0.001) (Fig. 3A and B). The correlation between HST-AHI and ODM-ODI was similar to night-to-night HST-AHI (Fig. 3A and B). Table 3 shows the diagnostic performance using the best Biologix-ODI cut-off for detecting mild, moderate, and severe OSA, as determined by HST. The AUC using ODI cut-offs of > 5, > 14, and > 25 events/h for the detection of mild, moderate and severe OSA, as detected by the ROC curve, were 0.983, 0.986, and 0.990, respectively (Fig. 4).

**Table 1:** Descriptive characteristics of the patients in the task force and study sample.

Variables	Population	Study sample
Age (years)	58.3 ± 13.0	56.7 ± 13.1
BMI (kg/m^2^)	31.8 ± 6.2	31.9 ± 6.3
Female, number (%)	563 (56.8)	280 (58.7)
**Race/color**		
White, n (%)	456 (45.9)	228 (47.8)
Black, n (%)	114 (11.5)	62 (13.0)
Brown, n (%)	340 (34.2)	165 (34.6)
Others, n (%)	23 (2.3)	7 (1.5)
No answer, n (%)	61 (6.1)	15 (3.1)
ESS	11.5 ± 6.1	10.3 ± 6.7
AHI, events/h	25.5 ± 24.9	24.7 ± 24.7.3
Minimal saturation, %	79.5 ± 7.0	80.2 ± 7.2
Mild/moderate/SEVERE OSA, %	28.0/24.4/30.1	30.9/21.9/29.7
**Smokers**		
Non-smokers, n (%)	599 (60.3)	315 (66.0)
Smokers, n (%)	85 (8.6)	38 (8.0)
Ex-smokers, n (%)	265 (26.7)	115 (24.1)
No answer, n (%)	45 (4.5)	9 (1.9)
Hypertension, n (%)	538 (54.1)	256 (53.7)
Diabetes, n (%)	260 (26.2)	125 (26.2)
Obesity, n (%)	583 (58.7)	290 (60.8)
COPD, n (%)	99 (10.0)	47 (9.9)
Dyslipidemia, n (%)	332 (33.4)	166 (34.8)
Coronary disease, n (%)	93 (9.4)	43 (9.0)

*BMI* body mass index, *COPD* chronic obstructive pulmonary disease, *ESS* epworth sleepiness scale, *AHI* apnea–hypopnea index.

**Table 2:** Comparison between HST performed in two nights in 62 patients.

Variables	Night 1	Night 2	*P*
Total register Time, minutes	437.0 ± 111.2	401.5 ± 82.0	0.068
HST-AHI, events/h	23.6 ± 21.4	23.4 ± 21.1	0.854
HST-ODI, events/h	22.9 ± 21.2	22.2 ± 20.9	0.572
Mean saturation %	92.7 ± 2.2	90.8 ± 12.0	0.016
Minimal saturation %	80.1 ± 7.1	79.6 ± 7.6	0.222

*HST* home sleep test, *AHI* apnea–hypopnea index, *ODM* overnight digital monitoring, *ODI* oxygen desaturation index.

**Table 3:** Diagnostic performance of ODM-ODI using the best cut-off for detecting mild, moderate, and severe OSA diagnosed by HST.

	Mild (AHI 5–15 eV/h)	Moderate (AHI 15–30 eV/h)	Severe (AHI > 30 eV/h)
ODM-ODI cut-off, ev/h	5	14	25
AUC	0.983	0.986	0.990
Sensitivity (%)	94.7	94.8	95.8
Specificity (%)	92.8	93.9	94.3
Accuracy (%)	94.3	94.3	94.8
PPV (%)	98.4	94.4	87.7
NPV (%)	78.6	94.3	98.1
LR+	13.1	15.5	16.9
LR−	0.1	0.1	0.04

*HST* home sleep test, *AHI* apnea–hypopnea index, *ODM* overnight digital monitoring, *ODI* oxygen desaturation index, *AUC* area under the curve, *PPV* positive predictive value, *NPV* negative predictive value, *LR*+ positive likelihood ratio, *LR−* negative likelihood ratio, *ev/h* events/h.

## Discussion

Our study showed that ODM using a high-resolution wireless oximeter and automated cloud algorithm for the detection of desaturations (Biologix) is an accurate method for OSA diagnosis and determination of OSA severity at home among patients with suspected OSA. The derived evidence from the observation of a good performance to detect at least mild, moderate, and severe OSA, as evidenced by the high AUC determined by the ROC curve (Fig. 4), high sensitivity, specificity, PPV, NPV, LR+ , and LM− (Table 3). Finally, the variability between HST-AHI and ODM-ODI was lower than that between night-to-night HST-AHI variability (Fig. 3).

PSG is considered the gold standard for diagnosing OSA^17^. However, HST has been widely validated for OSA diagnosis and management^15,17,18^. Despite the intended movement to enhance diagnostic accessibility, there remains an unacceptable level of OSA under diagnosis^10,15^. Overnight oximetry has long been advocated as an attractive technique for OSA diagnosis. However, a recent meta-analysis showed that the performance of oximeters designed for OSA diagnosis is extremely variable and depends on the study design and technology. The met-analysis found eight studies that compared PSG with ODM. However, only three of them performed simultaneous monitoring of PSG and ODM. The authors concluded that overnight oximetry was adequate to detect moderate to severe OSA when the 4% desaturation criterion was used^16^. We have previously shown a good performance of ODM-Biologix when compared to the sleep laboratory PSG-AHI^14^. Our study extends these findings by showing that ODM-Biologix has good accuracy for diagnosis at home for all severity ranges, including at least mild, moderate to severe, and severe OSA (Table 3). In contrast to our primary hypothesis that HST night-to-night variability would be similar to the HST-AHI versus ODM-ODI variability, we found a significantly lower variability of HST versus ODM than HST-AHI night-to-night variability (Fig. 3). The relatively high HST-AHI night-to-night variability in our study may be partly explained by the small sample size. However, a recent large sample of patients who repeated HST for three nights (10,340) showed that the confidence interval of night-to-night HST-AHI was approximately 21 events/h^16^, which is similar to the variability between HST-AHI and ODM-ODI found in our study (25.5 event/h). Similar levels of night-to-night variability have also been observed in sleep laboratory PSG^19^. Although in clinical practice, treatment decisions are made based on a single sleep study, the well-known night-to-night variability indicates that repeating sleep studies should be useful, particularly when there is a mismatch between clinical symptoms and sleep study results. However, this hypothesis has not been largely explored because of the inherent difficulties associated with repeating sleep studies. Therefore, simple, easy-to-use, and accurate techniques for OSA diagnosis that can be easily repeated, such as Biologix, may help to improve clinical decisions.

Biologix has several potential advantages that may help to unleash the OSA sub-diagnosis challenge. The cost of Biologix is one order of magnitude lower than that of HST equipment (less than 100 dollars vs. a few thousand dollars, respectively). HST studies have costs associated with disposable materials, including batteries and nasal cannulas, which are absent in the Biologix methodology. The HST requires specialized sleep centers and staff to analyze and interpret the HST study. In contrast, the Biologix platform automatically analyzed the data. Taken together, Biologix enables a paradigm shift that facilitates direct access to OSA diagnosis by non-sleep specialized professionals. This methodology may help to enforce programs for sleep apnea diagnosis and treatment directly at primary care centers^20,21^.

Although we considered HST-AHI as the gold standard method in this study, the concept that AHI is the single most important parameter for defining OSA severity has been questioned^22^. For instance, there is growing evidence that parameters derived from the oximeter, such as the ODI and the hypoxic burden, are more relevant than the AHI to determine the association with hypertension and to predict future cardiovascular events^23^. It must also be stressed that the treatment decision is dependent on the patients’ symptoms and the presence of comorbidities. Finally, the treatment decision will always depend on the center experience as well as on the patient’s willingness to accept specific treatment modalities, such as surgery or CPAP. Therefore, the present evidence of a good correlation between HST-AHI and ODM-ODI is robust proof that the Biologix platform provides sufficient information for clinical treatment decisions among patients with suspected OSA. In addition, the fact that Biologix is low-cost, minimally invasive, and accurate, leads to the possibility of multiple studies. Biologix can address the intrinsic uncertainty derived from night-to-night variability, which is largely ignored in clinical practice because of the economic and logistic difficulties of performing multiple sleep studies. Repeated sleep studies may also address the OSA severity fluctuations associated with alcohol intake. The device may also be particularly useful to follow up patients under different treatments, such as during titration of mandibular advancement devices, weight loss, and alternative treatments.

Our study had several limitations. First, Biologix was not offered to all patients who participated in this fast-track OSA diagnosis due to logistic limitations. However, we studied a large sample of patients with suspected OSA (Table 1). Second, ODM is dependent on smartphones with Internet connections, which may not be readily available in some areas. Third, the number of patients used on HST night-to-night variability was smaller than that of patients who used Biologix and HST. However, our night-to-night OSA severity variability is in line with previous studies^19,24^. Fourth, in this study compared ODM with HST considered as the gold standard. However, HST has potential limitations because it does detect sleep, sleep stages and arousals from sleep that may be relevant parameters in sub groups of OSA patients. Finally, ODM does not distinguish between central and obstructive events and has not been validated among patients with significant comorbidities, such as heart failure and severe pulmonary diseases.

In conclusion, ODM-Biologix is a simple method that has good performance for OSA diagnosis at home among patients with suspected OSA. As technology advances, new perspectives must be explored, such as the inclusion of more sophisticated analysis of pulse rate signal and sleep architecture using machine-learning algorithms.

## Data Availability

The datasets generated and/or analyzed during the current study are not publicly available due to the present report is part of a large project and involves other unpublished ancillary studies that are currently under analysis, but are available from the corresponding author on reasonable request.
